# Design of a force-measuring setup for colorectal compression anastomosis and first ex-vivo results

**DOI:** 10.1007/s11548-021-02371-8

**Published:** 2021-04-23

**Authors:** Jana Steger, Isabella Patzke, Maximilian Berlet, Stefanie Ficht, Markus Eblenkamp, Petra Mela, Dirk Wilhelm

**Affiliations:** 1 Research Group Minimally Invasive Interdisciplinary Therapeutical Intervention (MITI), Klinikum Rechts der Isar of Technical University of Munich, Munich, Germany; 2 Chair of Medical Materials and Implants, Department of Mechanical Engineering and Munich School of BioEngineering, Technical University of Munich, Munich, Germany; 3 Clinic and Policlinic for Surgery, Faculty of Medicine, Klinikum Rechts der Isar of Technical University of Munich, Munich, Germany

**Keywords:** Piercing, Colon, Forces, Endoscopy, Anastomosis, Implants

## Abstract

**Purpose:**

The introduction of novel endoscopic instruments is essential to reduce trauma in visceral surgery. However, endoscopic device development is hampered by challenges in respecting the dimensional restrictions, due to the narrow access route, and by achieving adequate force transmission. As the overall goal of our research is the development of a patient adaptable, endoscopic anastomosis manipulator, biomechanical and size-related characterization of gastrointestinal organs are needed to determine technical requirements and thresholds to define functional design and load-compatible dimensioning of devices.

**Methods:**

We built an experimental setup to measure colon tissue compression piercing forces. We tested 54 parameter sets, including variations of three tissue fixation configurations, three piercing body configurations (four, eight, twelve spikes) and insertion trajectories of constant velocities (5 mms^−1^, 10 mms^−1^,15 mms^−1^) and constant accelerations (5 mms^−2^, 10 mms^−2^, 15 mms^−2^) each in 5 samples. Furthermore, anatomical parameters (lumen diameter, tissue thickness) were recorded.

**Results:**

There was no statistically significant difference in insertion forces neither between the trajectory groups, nor for variation of tissue fixation configurations. However, we observed a statistically significant increase in insertion forces for increasing number of spikes. The maximum mean peak forces for four, eight and twelve spikes were 6.4 ± 1.5 N, 13.6 ± 1.4 N and 21.7 ± 5.8 N, respectively. The 5th percentile of specimen lumen diameters and pierced tissue thickness were 24.1 mm and 2.8 mm, and the 95th percentiles 40.1 mm and 4.8 mm, respectively.

**Conclusion:**

The setup enabled reliable biomechanical characterization of colon material, on the base of which design specifications for an endoscopic anastomosis device were derived. The axial implant closure unit must enable axial force transmission of at least 28 N (22 ± 6 N). Implant and applicator diameters must cover a range between 24 and 40 mm, and the implant gap, compressing anastomosed tissue, between 2 and 5 mm.

## Introduction

Since the last 15 years, current research efforts focus on enabling endoscopic interventions not only within the access lumen itself, but also on other organs of the abdominal cavity. Particularly interesting are interventions of daily clinical routine, such as the reconnection of bowel endings after colorectal resection. The indications are manifold (colorectal cancer, inflammatory disease, etc.) and it can be assumed that more than 1 million individuals have to undergo colorectal resection every year [1, 2]. Currently, for wide parts of the colon, anastomosis creation is the procedure step, which limits a further reduction of trauma the most. To achieve a smooth healing process, a direct serosa apposition of inverting end-to-end anastomoses is deemed necessary [3].

To securely connect two bowel endings and to prevent slippage, we are currently developing a compression-based implant which uses piercing of the bowel in the compression zone. However, endoscopic device development is hampered by the restrictions of limited space available and high functional demands. Force transmission to the endoscope tip is hereby a particular challenge. Accordingly, biomechanical characterization of gastrointestinal organs is needed to determine technical requirements and thresholds that define the functional design and load-compatible dimensioning. This also includes the assessment of size-related anatomical properties for dimensioning scalability with respect to lumen diameter and bowel wall thickness. Focus of the present study was to develop a system to reliably assess tissue penetration forces and the investigation of geometrical characteristics. By application of this innovative test rig, we were able to examine various implant- and applicator-related design specifications and their impact on puncturing forces.

## State of the art

The mechanical characterization of biological tissue is a central focus of various research studies. Potential applications include the development of force feedback applications or VR-based surgical simulators, the invention of new instruments or the input to in silico approaches for the investigation of surgical procedures, devices and therapeutic outcome prognosis [4]. Biomechanical computation holds the great potential of flexibility, time efficiency and the reduction, refinement and replacement of animal experiments in biomedical research [4].

Common experimental setups to derive the relevant parameters include uni- and biaxial tensile or indentation tests. Hereby, diverse research groups focus on the biomechanical characterization of porcine or human gastrointestinal tissues.

As we are interested in tissue puncture tests, related experimental setups will be primarily examined in the following. Typical research foci include electrode implantation into the brain or investigations of mechanical tissue interaction during single-body insertion.

Heijnsdijk et al. measured the perforation forces for porcine large bowel, pinching the tissue between two opposingly arranged metal hemispheres with a diameter of 1.5 mm. They determined a mean force of 13.5 ± 3.7 N [5]. Research groups around Abolhassani, Okamura, Bao, Jiang, Frick and Butz examined single-needle insertion forces for different tissue types and process relevant parameters affecting the measurement values [6–12]. Typical setups consisted of 1 or 2 DOF testing machines to control insertion, and 6 DOF electronic force transducers [6, 7, 9, 10]. Factors to be considered during puncture experiments include the type of tissue [9, 10], tissue fixation [13] and pre-conditioning [11, 12], needle geometry (body thickness/length, tip geometry) [7, 9, 10], puncturing device insertion trajectory (comprising velocity, acceleration and rotation) [6, 9, 10] and interrupted or continuous insertion process [10].

Kwon et al. [14] developed the BMPM system (Biomaterial property measurement system) to enable measurement of deformation and puncture forces of esophageal, colonic and gastric tissues in vivo. However, experiments described were performed in vitro on porcine tissue. For piercing colon with a needle, a peak force of 0.056 N was measured.

For anastomotic device-tissue interaction, Schell came closest to answering our question with his investigations. In 2005, he developed a semicircular staple suture device for transanal endoscopic microsurgery [15]. For this purpose, the forces occurring during stapling with 17 clamps instead of 24 were measured. The force ratio from handle to staple magazine was specified to be 9.3. A force of 202 N was obtained at the lever and 1885 N at the stapler head. However, there was no specification of the experimental setup given. As the other groups’ experiments comprised only investigations with one single puncturing body and as we need to provide more penetration points for safe and non-slip positioning of the tissue in the anastomosis zone, the results are not sufficient for the design and dimensioning of an endoluminal applicator.

Therefore, the purpose of the present study was to investigate various implant- and applicator-related design specifications concerning tissue fixation to the applicator and tissue puncturing by the implant. We investigated whether different fixation point arrangements, amounts of tips, as well as varying insertion speeds and accelerations of the implant influence insertion force and process.

For our experiments, we chose porcine tissue, as the physiology and anatomy (including length) of the pig’s gastrointestinal tract, as well as its digestive function, blood flow characteristics and tissue mechanics have a very close resemblance to those of an adult human [16, 17]. Heijnsdijk et al. [5] compared perforation forces of human and pig small bowel and didn’t detect any difference. Kararli assessed the gastrointestinal anatomy, physiology and biochemistry of humans in comparison with commonly used laboratory animals and suggested to use porcine tissue for colon-related experiments [18]. Variations of anatomy are present with respect to the configuration of the colon. Pigs have a short transverse and a descending colon, but no sigmoid flexure. Furthermore, the cecum and the colon ascendens are coiled together and loops adhere to each other in the spiral colon. However, these differences in anatomy didn’t have any influence on our methods as we performed our experiments with explanted samples. Due to the high degree of resemblance, we decided to define the design specification for our system, based on the experimental results, without any further transformation.

## Material and methods

**Fig. 1:**
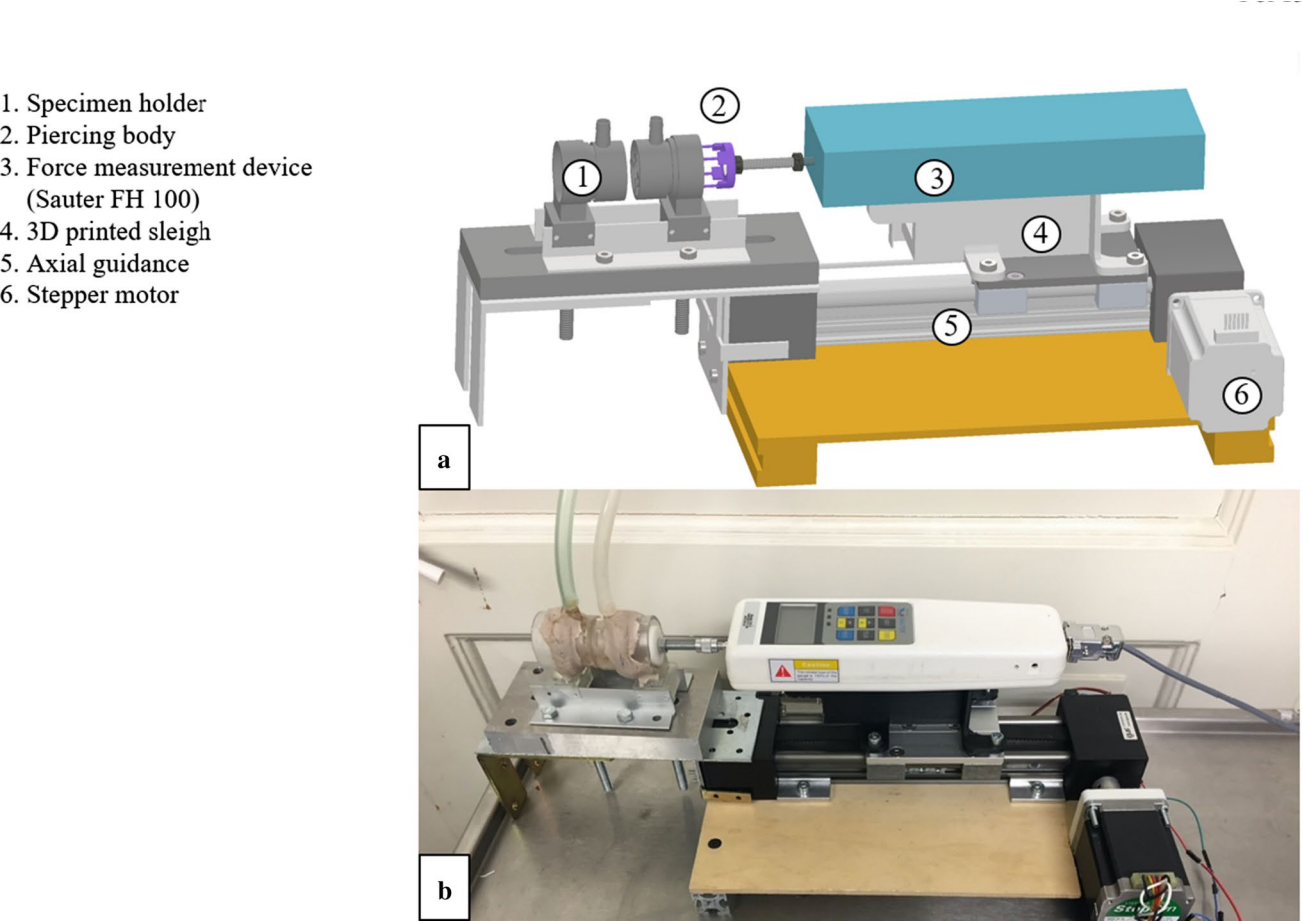
**a** CAD model of experimental setup with two tissue fixation entities (applicator dummies) (1) a disc with spikes (2) a Sauter, FH 100 force gauge (3) fixed on a slide (4), which is movable translationally (5). The movement was driven by a stepper motor (Sanyo Denki, StepSyn Type 103H7126-0740) (6). **b** Photo of the experimental setup

**Fig. 2:**
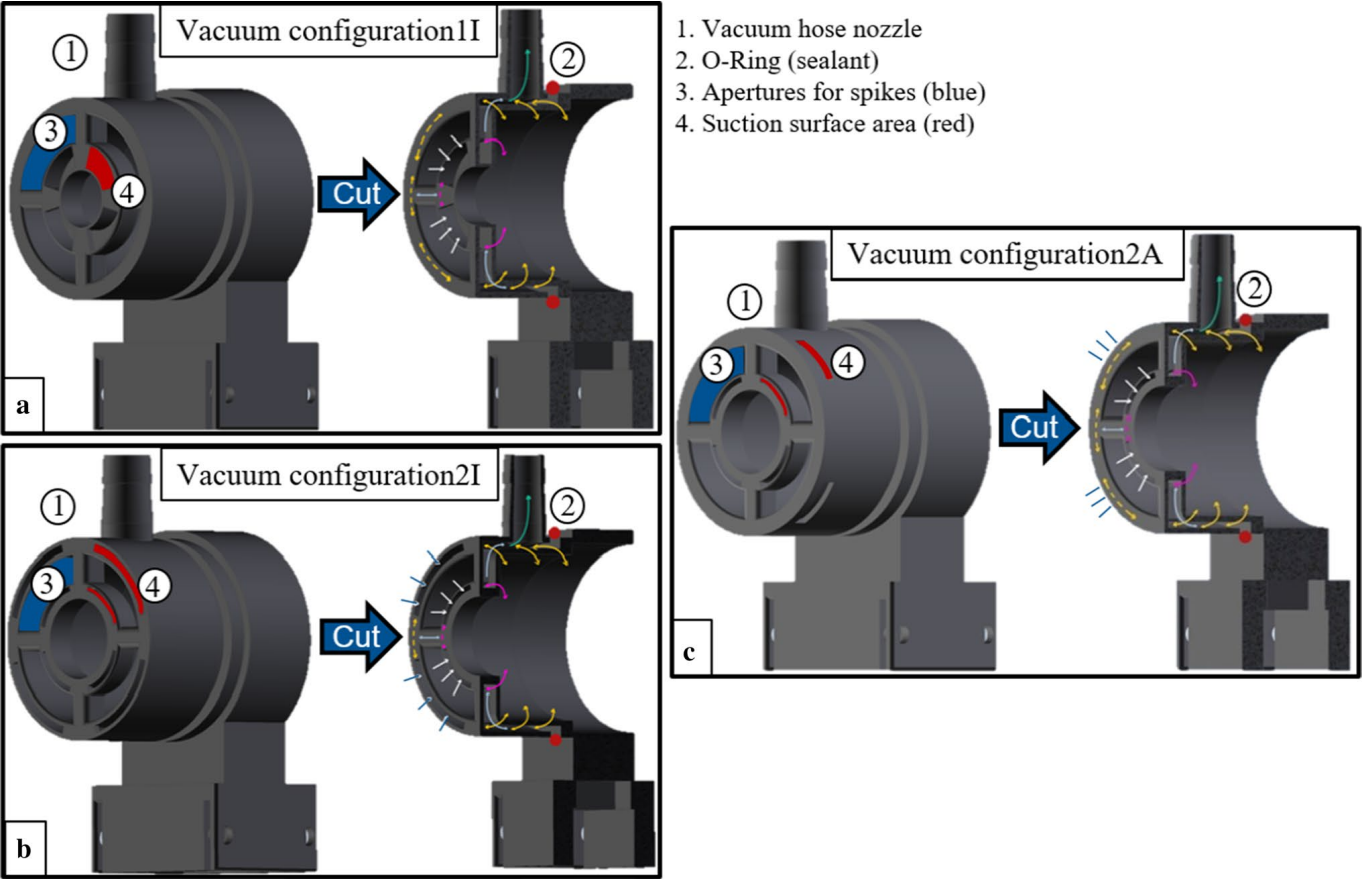
Illustration of the fixation entities with variation in suction surface arrangement (red area 4). Spikes are pushed through blue marked areas (3). **a** Vac1I: suction surfaces exclusively in the center, **b** Vac2I: suction surfaces at frontal face inside and outside of the spike openings. **c** Vac2A: suction surfaces at frontal face in the center and on the sheath. Vertical cuts through the applicator dummies on the right side of each image, visualizing the hollow wall structure for circular air flow, marked by arrows, to (blue/white arrows) and within (yellow/pink arrows) the fixation entities, occurring as soon as the vacuum pumps are connected to the nozzle (1) and switched on (green arrow)

**Fig. 3:**
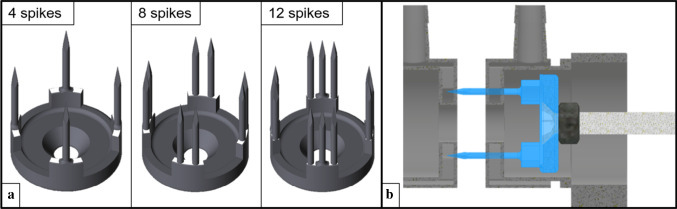
**a** Piercing bodies with different amounts of spikes (four, eight, twelve). For configurations with four spikes, the needles were applied centrally on the segment, for eight spikes, they were positioned offset to the center with a distance of 18° in between, for twelve spikes, one tip centrally with two others in 14° distance to both sides, **b** vertical cut through applicator and implant dummies during piercing process

**Fig. 4:**
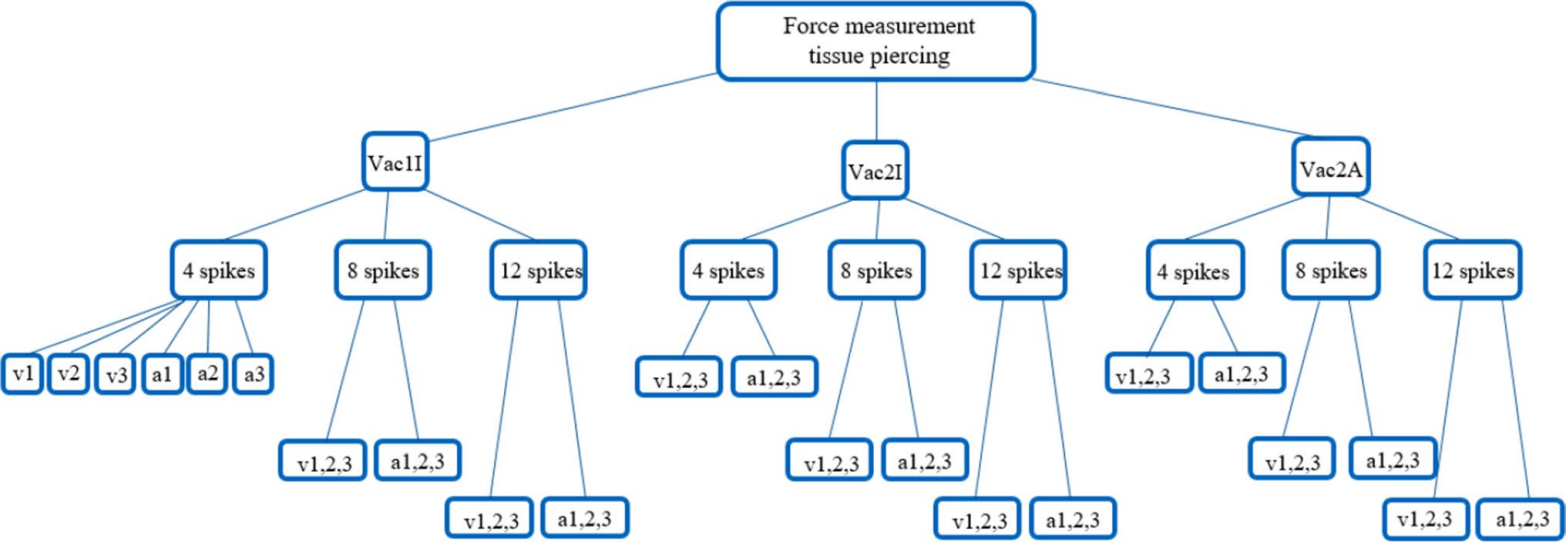
Overview of 54 tested parameter sets, with each *n* = 5 samples ($n_{{{\text{total}}}}$ = 270). A total of three different vacuum configurations, three amounts of spikes and six trajectories with constant velocities and accelerations were tested

**Fig. 5:**
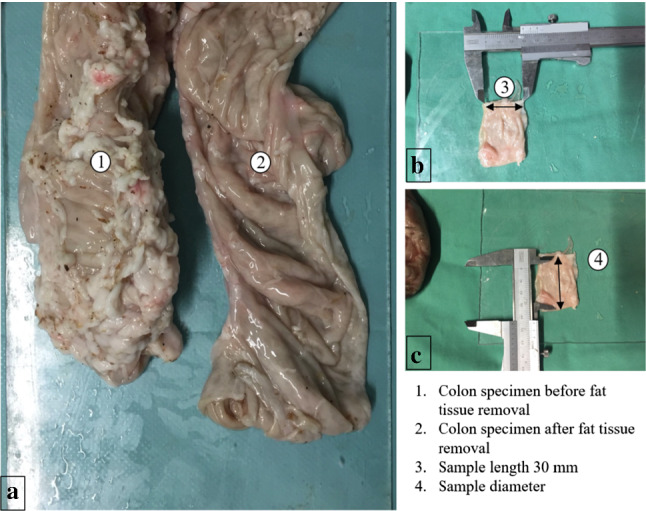
**a** Pig colon (1) before and (2) after removal of fat tissue. **b** Preparation of ~ 30 mm long, tubular samples. **c** Measurement of half of sample circumference for each specimen to calculate sample diameters

**Fig. 6:**
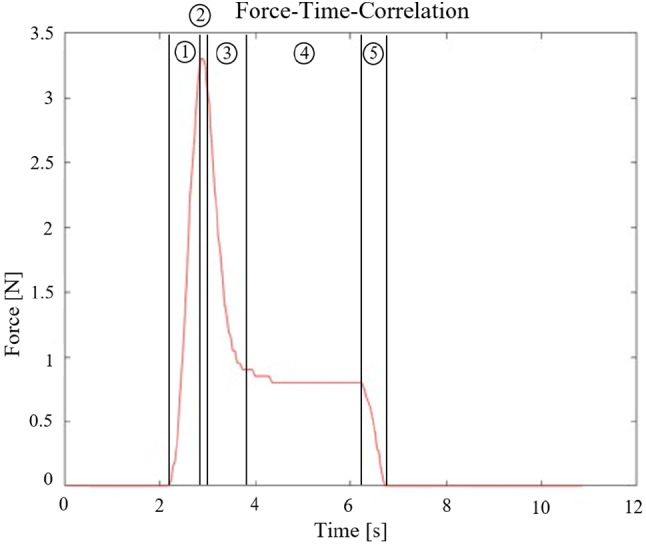
Specific force–time-correlation. Force measurement starts with initial tissue contact. Force rises rapidly (1) to the peak force (2), and just as abruptly thereafter falls back (3) to a plateau (4) until force finally drops back to zero (5)

Our experimental setup consisted of two identical tissue fixation entities (applicator dummies) mounted on a centering rail (Fig. 1a-1) facing each other in 4 mm distance, a disc with segmental, symmetrically arranged spikes (implant dummy) (Fig. 1a-2), and a Sauter, FH 100, calibrated force gauge (Fig. 1a-3). The FH 100 was fixed onto a slide (Fig. 1a-4) movable translationally (1DOF) along a guide rail (Fig. 1a-5). The movement was enabled by a wedge toothed belt tensioned onto two gears, of which one was driven by a stepper motor (Sanyo Denki, StepSyn Type 103H7126-0740) (Fig. 1a-6). An Arduino (Arduino Uno) with a motorshield (Arduino Motor shield Rev3) was used to control motor speed and acceleration, thus $\frac{{{\text{steps}}}}{{{\text{min}}}}$. To ensure sufficient power supply, an external power source was connected and powered with 5 V. The piercing body was fixed to the force gauge (Fig. 1).

### Tissue fixation

Each specimen holder featured openings through which the implant tips were passed and suction surfaces to atraumatically fix the tissue using vacuum [19]. The sample holder had a diameter of 35 mm and the total suction area of all entities was ~ 130 mm^2^. Connecting a vacuum pump to each of the specimen holders, with a maximum achievable negative pressure of 80 kPa with respect to atmospheric pressure, the fixation force per side was $F_{{{\text{fixation}}}} = \Delta p*A = 10.4 \,{\text{N}}$ ($\Delta p$:Difference between atmospheric and system pressure; A: surface of suction area). Three variations of suction surface configurations (Fig. 2) were investigated to find out whether the number and arrangement of tissue fixation points effects the penetration process/force. Vacuum configuration1 (Vac1I) featured suction surfaces exclusively in the center. For vacuum configurations2 (Vac2I), suction surfaces were split and located to both sides of the frontal spike apertures, to firmly stretch the tissue over the openings. The same effect was intended for configuration3 (Vac2A), for which suction surfaces were located toward the lumen center at frontal face and on the sheath.

### Spikes

The implants, with a diameter of 26.5 mm, comprised four equidistant segments arranged symmetrically on a plate claiming each 36° of the needle ring. Along these fixed segments, one to three tips were applied, resulting in configurations with four, eight and twelve tips. The podiums ensured sufficient needle length for piercing two tissue layers, when advancing from the first into the second specimen holder (Fig. 3b). On these platforms, the spikes were arranged symmetrically to the center (Fig. 3a). The tip geometry was designed according to Okamura et al., who detected lowest forces for triangular/tetrahedral tip shape [7] (Fig. 3a). We used needles with a tip angle of 15°, a length of 20 mm and a diameter of 1.7 mm. The needles had to be of sufficient length to compensate for indentation that typically occurs in biological tissue due to viscoelastic properties. All piercing bodies and specimen holders were 3D-printed using Formlabs2 from standard White^1^ or Clear resin.^2^

### Trajectory

We specified trajectory velocities and accelerations in orientation to Abolhassani et al. [6]. By variation of motor velocity and acceleration, three constant axial velocities of $$v_{1} = $$ 5 mms^−1^, $$v_{2} =$$ 10 mms^−1^ and $$v_{3} =$$ 15 mms^−1^ and three constant axial accelerations of $$a_{1} = $$ 5 mms^−2^ $$ a_{2} =$$ 10 mms^−2^ *a*_3_ = 15 mms^−2^ were examined, for which final velocities of 12.5 mms^−1^, 17.7 mms^−1^and 21.6 mms^−1^ were achieved when reaching the piercing plane. The corresponding Arduino script motor velocities were 4 rpms^−1^, 8 rpms^−1^ and 12 rpms^−1^, and the motor accelerations 10 rpms^−2^, 100 rpms^−2^ and 150 rpms^−2^.

Figure 4 provides an overview over all parameter sets assessed within our study. For each parameter set, experiments with *n* = 5 samples were performed.

### Sample preparation

Pig colons were obtained day-fresh from the local butcher, cooled and kept moist during the day until use. Fat tissue was removed (Fig. 5a) to realistically simulate bowel margins readily prepared for anastomosis closure. For the experiments, colon was cut perpendicular to the Taenia Libera into approximately 30 mm long tubular segments (Fig. 5b). For each parameter set, five sample pairs were prepared and stored in two kidney shells covered with saline (NaCl 0.9) [20] soaked bandages, numbered according to the sequence in the experimental procedure. The remaining prepared specimens were stored in a cool box.

Each specimen couple was assigned an identification code (ID) comprising: [vacuum configuration]_[number of spikes] _[velocity value/acceleration_value] _[Trajectory specification]_[sample number within parameter set] ([1I/2I/2A]__[4/8/12]_[5/10/15]_[v/a]_[1/2/3/4/5]), by which the recorded characteristics diameter and tissue thickness were correlated with the measured piercing forces. For example, sample three for Vac2I, pierced with eight tips and a constant trajectory velocity of 15 mms^−1^ had ID: 2I815v3.

Before the experiment, each specimen was draped flat on a base plate to measure half of the circumference, $u_{0.5} = \frac{U}{2}$, in the center of each probe (Fig. 5c) and respective diameter was calculated by $D = 2*\frac{{u_{0.5} }}{\pi }$, subsequently. The pierced tissue thickness of each specimen pair was assessed by means of indirect optical measurement.

### Experiments

Prior to each parameter set, a script specifying the trajectory was uploaded to the Arduino board. One of the prepared bowel segments was mounted onto each of the specimen holders (Fig. 1), so that the fixation surfaces were inside the sample lumen. The open bowel ends were formed into sausage-endings and adjusted around the circumference until all suction apertures were covered. Switching on the vacuum pumps, a uniform negative pressure established on both sides. Using a MATLAB script, the compression forces occurring during tissue piercing measured with the FH 100 were recorded over time. After each trial, the specimens were replaced.

### Statistical analysis

To investigate the influences of spike amount variations, different fixation point arrangements and trajectories, we statistically analyzed our results. Therefore, the Gaussian distribution of each parametric set was checked with the Shapiro–Wilk test based on a significance level of $$\alpha_{N} =$$ 0.05. A parametric three-way ANOVA (A), as well as the non-parametric KRUSKAL–WALLIS (KW) test were used, whereby $$H_{0} $$ hypotheses were rejected with respect to a significance level of $$ \alpha_{A/KW} = 0.01667 $$ for both tests.

## Results

### Force–time–correlation

For each experiment, occurring forces were plotted over time (Fig. 6). This correlation is qualitatively equal for all vacuum configurations, peak numbers, and trajectory specifications. As soon as tissue penetration begins, the force rises sharply (1) resulting in the peak force (2), and just as abruptly thereafter falls back (3) to a plateau (4) until force finally drops back to zero (5). A qualitative difference was observed in the plateau slope, which was not always horizontal, but sometimes showed a decrease in force over time.

### Maximum and minimum forces

The mean (*n* = 5) and single measurement maximum and minimum peak forces for all implant configurations (four, eight, twelve spikes), over all assessed parameters, were evaluated and reported in Table 1.

**Table 1:** Mean and single value maximum and minimum peak piercing forces

Peak piercing forces	Mean maximum		Overall maximum		Mean minimum		Overall minimum	
Implant configuration	ID	Force (N)	ID	Force (N)	ID	Force (N)	ID	Force (N)
4	2I10v	6.4 ± 1.5	2I10v_2	8.7	2A15a	3.2 ± 0.3	2A15a_5	2.9
8	2A5a	13.7 ± 1.4	2I5v_1	16.8	2I10a	8.7 ± 0.6	2I15a_3	5.0
12	2I15v	21.7 ± 5.8	1I15v_5	30.4	2I15a	10.3 ± 1.7	2I15a_3	9.0

### Statistical analysis

Seven out of 54 parameter sets were not normally distributed. Therefore, non-parametric and parametric tests were used for the statistical analysis.

### Trajectories

**Fig. 7:**
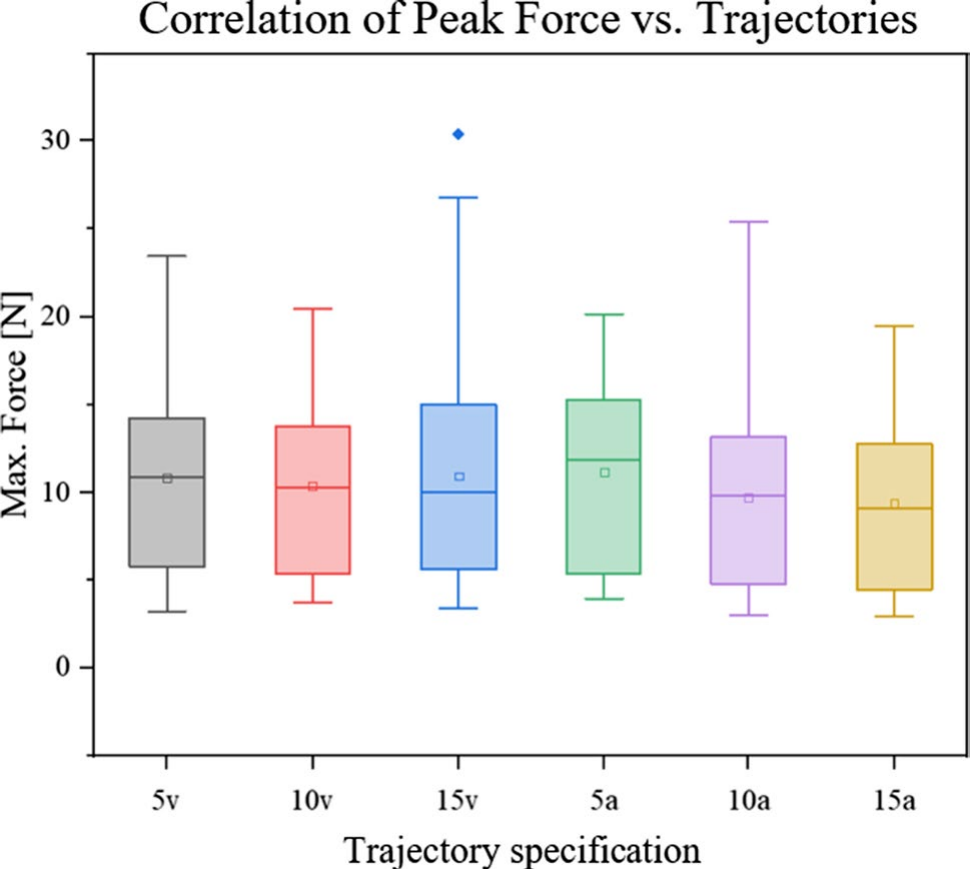
There was no statistically significant difference of insertion forces between all groups $v_{1}$, $v_{2}$, $v_{3}$, $a_{1}$, $a_{2}$ and $a_{3}$. (1I mean = 10.9 ± 5.8 N; median = 10.15 N), (2I mean = 10.0 ± 4.2 N; median = 9.8 N), (2A mean = 10.4 ± 5.3 N; median = 10.7 N)

To identify possible differences between tested velocities and accelerations, our first hypothesis was: $H_{0} :$ There is no difference in occurring insertion forces for groups $v_{1}$, $v_{2}$, $v_{3}$, $a_{1}$, $a_{2}$ and $a_{3}$. This assumption was confirmed with a *p* value_A_ of 0.513 and a *p* value_KW_ of 0.2362, thus there was no statistically significant difference between the groups (Fig. 7).

### Vacuum configuration

**Fig. 8:**
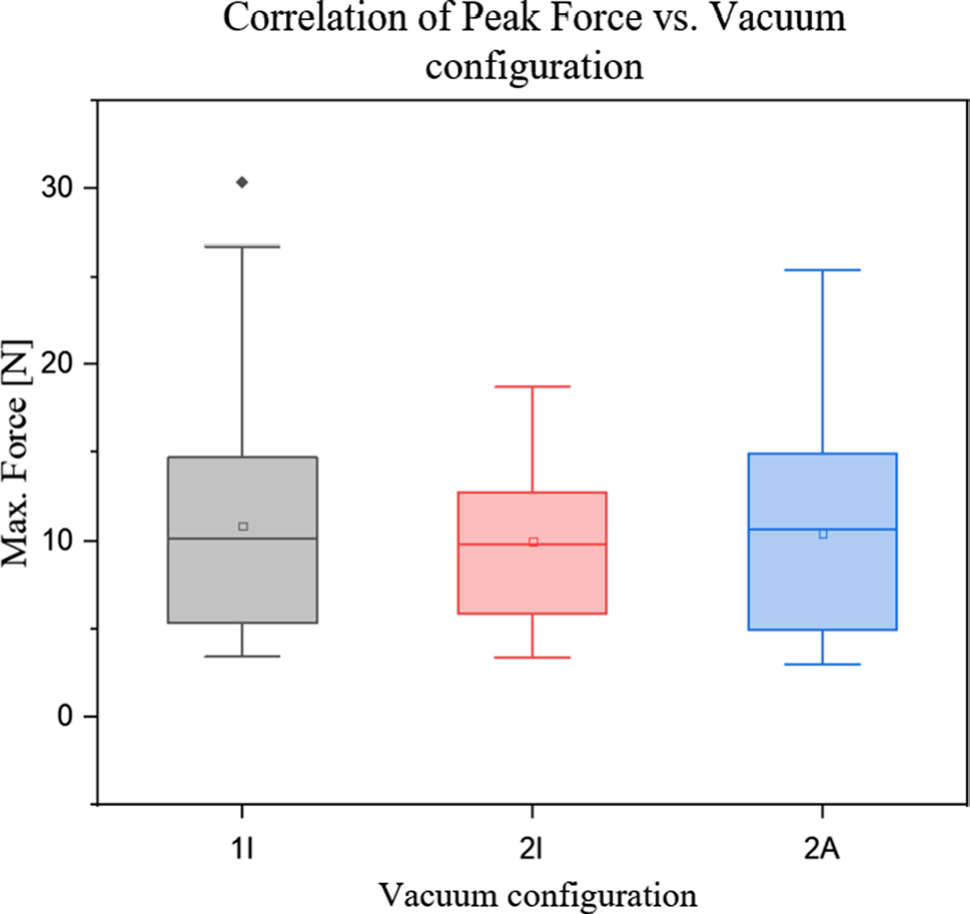
There was no statistically significant difference between insertion forces for different tissue fixation point arrangements. (1I mean = 10.9 ± 5.8 N; median = 10.15 N), (2I mean = 10.0 ± 4.2 N; median = 9.8 N), (2A mean = 10.4 ± 5.3 N; median = 10.7 N)

Second hypothesis: $H_{0} :$ There is no difference in occurring insertion forces depending on the arrangement of fixation points (different vacuum configurations used). The ANOVA test revealed a *p* value_A_ of 0.501 and the KRUSKAL–WALLIS test a *p* value_KW_ of 0.845, thus there was no statistically significant difference for the three vacuum configurations (Fig. 8).

### Amount of spikes

**Fig. 9:**
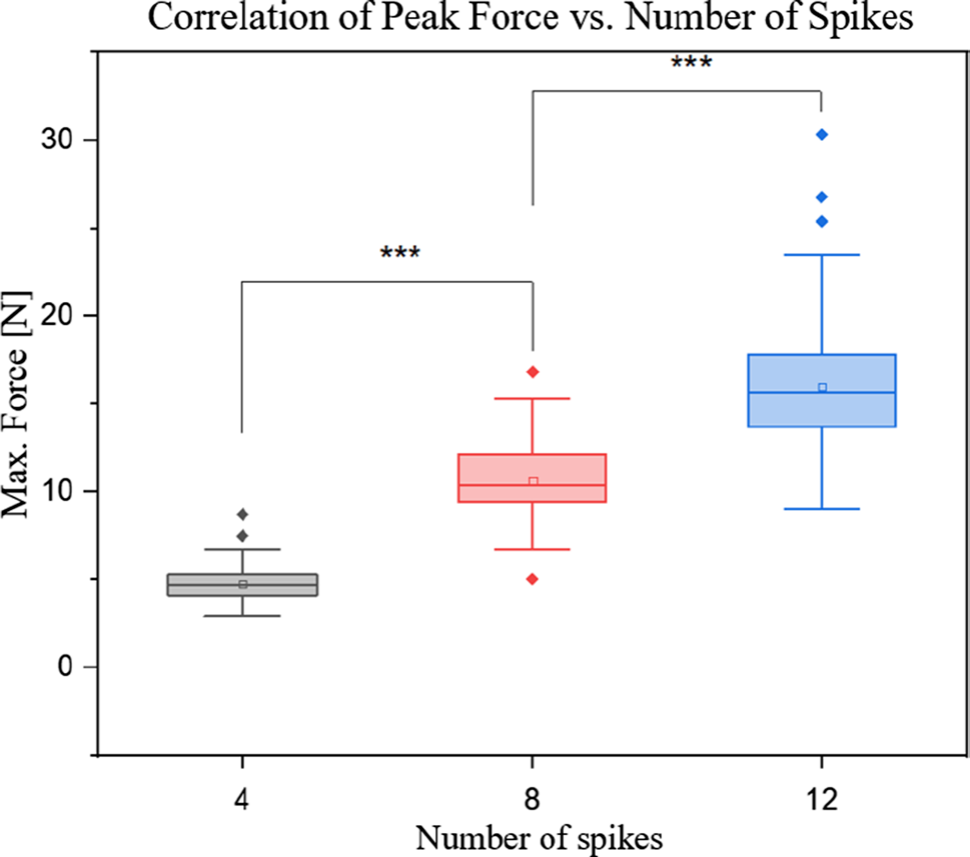
With *p* values_A/KW_ < 2.2e-16, there was a statistically significant increase in maximum forces with increase of spike amount for both, parametric and non-parametric tests. (4 spikes: mean = 4.7 ± 1.0 N; median = 4.7 N) (8 spikes: mean = 10.6 ± 2.0 N; median = 10.3 N) (12 spikes: mean = 15.9 ± 13.5 N; median = 15.7 N)

Third hypothesis: $H_{0} :$ There is no difference in occurring insertion forces depending on the amount of spikes (4, 8, 12 spikes). With *p* values_A/KW_ of < 2e-16 for ANOVA and the KRUSKAL–WALLIS test, this hypothesis was rejected with respect to $\alpha_{{\text{A/KW}}} = 0.01667.$ The insertion force measured increased significantly with an increasing number of implant spikes (Fig. 9).

**Fig. 10:**
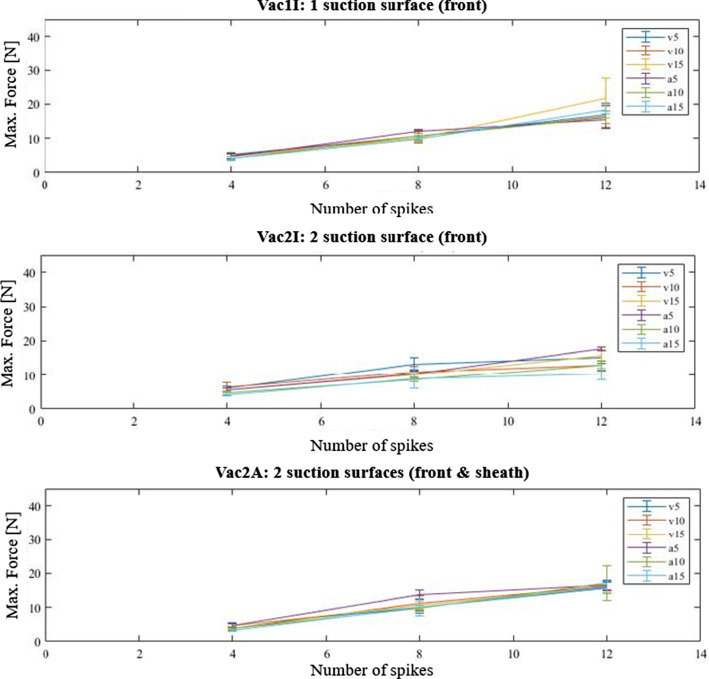
Correlation between mean values of insertion forces (for each trajectory and vacuum configuration) and increasing spike amount. The errorbars indicate the standard deviation of each data set

Plotting the correlation between the mean value of peak insertion forces and the number of spikes, we observed a consistent coherency for all velocities, accelerations and vacuum configurations (Fig. 10).

### Diameter and thickness of pierced tissue

We evaluated a mean diameter of 31.3** ± **4.6 mm (*n* = 540), and a mean pierced tissue thickness of 3.8** ± **0.7 mm (*n* = 270). The 5th and 95th percentiles, $$x_{p = 0.05}$$ and $$ x_{p = 0.95}$$ were calculated by $$x_{p} = 0.5*\left( {x_{n*p} + x_{n*p + 1} } \right)$$. The 5th percentile for the lumen diameter is $$x_{0.5}^{{{\text{diameter}}}} = 24.1\,{\text{mm}}$$, and the 95th is $$x_{0,95}^{{{\text{diameter}}}} = 40.1\,{\text{mm}}$$. Same procedure revealed the 5th and 95th percentile for the pierced tissue thickness to be $$x_{0.5}^{{{\text{thickness}}}} = 2.8\,{\text{ mm}}$$, and $$x_{0,95}^{{{\text{thickness}}}} = 4.8\,{\text{mm}}$$, respectively.

## Discussion

Improving colorectal surgery is of particular interest, due to the high numbers of patients affected every year [1, 2]. To reduce trauma in clinical daily life, new instruments are of particular interest, as for example innovative anastomotic devices. However, there are few studies characterizing the mechanical tissue-system interaction, data, which would be relevant for dimensioning and design of such instruments. It was our goal, to fill this gap, give orientation values and provide general design and dimensioning guidelines for new instruments and colorectal anastomosis system development.

### Force–time-correlation

Profile phases are explained under consideration of other research groups’ findings. In contrast to Abolhassan et al. [6, 7, 9], we evaluated the force–time instead of the force–displacement correlation. Needle, thus tissue displacement and the time relate linearly to each other, which is why comparison of the curves is still intuitively possible. Phase (1) corresponds to indentation of viscoelastic tissue. Stress applied to the tissue, and therefore measured force, rises with increasing piercing body displacement, respectively, time. In our profile, this correlation looks linear. Comparing the results to Bao and Okamura, we observe polynomial curve progressions, which are described by second or third-order polynomial spring model [7, 9]. However, they used massive specimens (i.e., whole liver) instead of tissue slices. In a specimen block, subjacent tissue layers strongly influence the overall stiffness, which can therefore not be considered constant over entire insertion depth. The results of Dimaio and Abolhassani, based on experiments with thin tissue samples, are more comparable to ours. Dimaio used a homogeneous linear elastostatic model to predict forces during soft tissue puncture [21] and for Abolhassani the displacement-force curve approaches a linear progression as well. However, our slope is much higher than for 1:1 depiction of indentation, as the process itself, depending on the transversal speed, only takes a few miliseconds. The peak indicates the event of puncture [6, 7, 9] (2), occurring when the tissue stress, created by the piercing bodies, exceeds a critical threshold and micro-cracks propagate [22], resulting in tissue failure and an abrupt drop of force recorded (3). While for Abolhassani, the force remains approximately zero after the drop, it rises again for Bao and Okamura [7, 9], as frictional forces occur with further penetration into specimen block. At the plateau (4), the sleigh remains in its position for 3000 ms. Variations in the plateau slope may occur due to viscoelastic relaxation. In the last phase (5), the force drops back to zero as the sleigh is pulled back, out of the tissue.

## Trajectories

The situation was similar for the tested insertion trajectories, as our analyses did not show any statistically significant difference between the groups of $v_{1}$, $v_{2}$, $v_{3}$, $a_{1}$, $a_{2}$ and $a_{3}$. Considering $F = m*a$ and $F\Delta t = m*\Delta v$, this result does not match the initial expectation. This could be due to the fact that the differences between the tested groups were very small. For our results, trajectory specification within these ranges of piercing velocities and accelerations didn’t effect on the required piercing forces.

### Vacuum configuration

$H_{1}$ was based on the assumption that splitting the suction areas would stretch the tissue over the spike apertures reducing tissue indentation during piercing and making the process more independent of viscoelastic tissue property variations. We supposed an increase in tissue tension would in turn lead to higher piercing forces [11, 12]. However, with respect to our results, we saw, that the fixation point arrangement did not effect on the forces required to pierce the tissue.

### Piercing force and influence of implant configurations

We measured maximum mean peak forces for four, eight and twelve spikes, of 6.4 ± 1.5 N, 13.6 ± 1.4 N and 21.7 ± 5.8 N, respectively. Schell used 17 staple clamps to measure the forces, resulting in 34 insertion points. He detected 202 N at the handle and 1885 N at the stapler tip, which is more than 80 times higher than our highest mean peak force of 21.7 N. As the experimental setup was not specified, a comparison is hardly possible [15]. However, we assume that a large part of the applied force in this case was converted into plastic deformation of the staples and did not occur due to stapler-tissue interaction.

Statistical analysis showed a significant increase in insertion forces with an increase in amount of spikes (Fig. 9). With increasing number of insertion bodies, the characteristic spike-tissue interaction force of each single body accumulated. The characteristic profile phases (Fig. 7) observed for the insertion of a single needle [6, 7], as well as for the implant configurations with four, eight and twelve tips, are all qualitatively equal, with only difference in peak force height. Furthermore, since the tips were not distributed evenly over the entire circumference of the piercing body, but only within the segments, the density per segment increased with rising tip amount. Once the tips contact tissue surface, micro-injuries in the surface are created [22], acting as anchor points. As spikes are pushed forward, indentation occurs [6, 7, 21]. Tissue tension is therefore increased between individual tips/attachment points. This pre-tensioning effect may contribute to the increase in insertion force. This correlation was also discovered by Frick and Butz [11, 12]. Implanting electrodes into the cerebral cortex of rats, Jensen et al. also detected an increase in force with increasing number of electrode shafts inserted [23]. By plotting the dependence between the number of spikes and the maximum force, we observed that the slope is nearly equal for all vacuum configurations and trajectory specifications, which led us to the conclusion, that these parameters do not influence the coherency. The derived correlation can be used to optimize the closing mechanism and reduce compression forces required, as orientation values for alternative implant configurations can be interpolated.

However, the measured maximum mean peak forces already do serve as orientation benchmarks for the design of our endoscopic anastomosis implant closing unit. Based on this, a force transmission mechanism is selected and dimensioned accordingly. To ensure high anastomosis stability, we decided to design an implant with 12 tips, for now. Therefore, it will be necessary to enable an axial force transmission of at least 28 N (22 ± 6 N) at the endoscope tip.

### Wall thickness and lumen diameter

The colon lumen diameter limits the acceptable size of our application device and determines the required range of the implant expansion unit. The colonic wall thickness is of particular interest, to achieve an optimal compression pressure, thus providing sufficient stability without compromising blood supply of the bowel margins. The 5th and 95th percentiles, $x_{p}$, are usually taken for ergonomic dimensioning of products to cover the majority of a population of interest. These benchmarks give us preliminary orientation of an upper and lower limit to be addressed for the size adaptability of our system. The average colon diameter of humans is 5 cm, and therefore comparable to our results [18]**.** Based on our findings, we conclude that the diameters of implant and applicator will be in a range between 24 and 40 mm, and the implant gap, compressing anastomosed tissue, between 2 and 5 mm.

Higher velocities and accelerations, with larger variations between groups, and more complex implant geometries and closure mechanisms will be assessed in the next step.

## Footnotes

1 Formlabs: https://formlabs-media.formlabs.com/datasheets/1801089-TDS-ENUS-0P.pdf; last accessed:10.01.2020.

2 Formlabs; https://formlabs-media.formlabs.com/datasheets/Clear_Resin_Technical.pdlast accessed:10.01.2020.
